# Use of MRSA surveillance data for infection control: individual units rather than entire hospital as the basis for improvement

**DOI:** 10.1186/1753-6561-5-S6-P13

**Published:** 2011-06-29

**Authors:** P Gastmeier, F Schwab, I Chaberny, C Geffers

**Affiliations:** 1 Institute of Hygiene and Environmental Medicine, Berlin, Germany; 2 CHARITÉ – University Medicine Berlin, Berlin, Germany; 3 Institute of Medical Microbiology and Hospital Epidemiology, Hannover Medical School, Hannover, Germany

## Introduction / objectives

To analyze which surveillance system (a hospital based or a unit based) leads to a greater decrease in incidence density of nosocomial MRSA.

### Design

Two cohort studies of surveillance data.

### Setting

Two MRSA surveillance components exist within the German national nosocomial infection surveillance system KISS: one for the whole hospital (i.e. only hospital based data and no rates for individual units) and one for ICU-based data (rates for each individual ICU).

### Participants

Data from a total of 224 hospitals and 359 ICUs in the period from 2004 to 2009.

## Methods

Development over time was described first for both surveillance systems. In a second step only data were analyzed from those hospitals/ICUs with continuous participation for at least four years. Incidence rate ratios (IRR) with 95% confidence intervals were calculated to compare incidence densities between different time intervals.

## Results

In the baseline year the mean MRSA incidence density of hospital acquired MRSA cases was 0.25 and the mean incidence density of ICU-acquired MRSA was 1.25 per 1000 patient days. No decrease in hospital-acquired MRSA rates was found in a total of 111 hospitals with continuous participation in the hospital- based system. However, in 159 ICUs with continuous participation in the unit-based system, a significant decrease of 29 % in ICU-acquired MRSA was identified.

## Conclusion

A unit-based approach of surveillance and feedback seems to be more successful in decreasing nosocomial MRSA rates, compared to a hospital-based approach.

## Disclosure of interest

None declared.

